# Paediatric Femoral Diaphyseal Fractures in a South Wales Tertiary Centre: An Account of Trend in Management and Complications Over 16 Years

**DOI:** 10.7759/cureus.30917

**Published:** 2022-10-31

**Authors:** Rajiv P Doshi, Claire Carpenter

**Affiliations:** 1 Trauma and Orthopaedics, University Hospital of Wales, Cardiff, GBR

**Keywords:** femur and fracture, diaphyseal fractures, children's, paediatric orthopedics, femoral shaft fractures

## Abstract

Femoral shaft fractures in children have seen a number of interesting developments over the past 20 years. This is a retrospective cohort study looking into epidemiological and outcomes data of femoral shaft fractures in children treated at a tertiary centre in Wales from 2005-2021. Over a period of 16 years, there has been a significant increase in the number of rigid or elastic nailing and submuscular plating, coinciding with a dramatic reduction of external fixation for definitive treatment of diaphyseal femoral fractures. All patients above five years of age underwent operative fixation, with elastic or rigid intramedullary nailing the treatment of choice. Following multiple linear regression with 16 possible explanatory factors, this study found a statistically significant increase in time to union for open fractures, pre-operative translation, and operation time. Furthermore, there was a significant increase in post-operative leg length discrepancy for right versus left-sided fractures. Overall complication rates were 4% for minor and 8% for major complications. Complication rates were lowest for rigid intramedullary nailing and highest for external fixation. No cases of avascular necrosis were found for 27 rigid intramedullary nails inserted. Overall this study reports treatment choices and outcomes in keeping with current trends in management for paediatric femoral shaft fractures.

## Introduction

Femoral shaft fractures account for less than 2% of paediatric fractures [1], however, they continue to be associated with longer than average hospitalisation of children, periods of disability and lower limb asymmetry in the growing child [2,3]. Despite the multitude of methods that have been utilised to manage these fractures, they continue to pose a challenge for orthopaedic surgeons [4].

They have a bimodal distribution with peaks at ages two and 17 respectively, and are two and a half times more common in boys than girls [5,6]. They are the commonest long bone fractures associated with high-energy trauma in children with an incidence of 0.33-0.22/1000/year [7].

The majority of femoral shaft fractures in children are caused by motor vehicle collision, especially in the older children or adolescents, however, falls are the commonest cause in children less than six years of age [8]. As part of the high-energy nature of femoral shaft fractures, these children tend to be managed either in major trauma or tertiary centres. This study looks into the epidemiological and outcome data within the management of paediatric femoral shaft fractures in the Children’s Hospital for Wales, a tertiary centre established in 2005 that supports hospitals in South Wales in paediatric patient care and services.

## Materials and methods

This study was a retrospective observational study of paediatric femoral diaphyseal fractures in the Children’s Hospital for Wales (CHfW). Patients under 16 years of age who underwent treatment in CHfW from 2005-2021 were identified via the hospital database. Patient demographics were collected including age at injury, gender, and date of injury. Fracture details were collected including site, side, pattern, open/closed, polytrauma, and primary procedure. Radiographic data collected included pre-operative (pre-op) fracture angulation in anteroposterior (AP) and sagittal planes, percentage translation, comminution, and amount of shortening. Details of any complications were also obtained including paediatric intensive care unit (PICU) stay, repeat procedures, superficial or deep infections, problems with wound or bone healing, avascular necrosis (AVN), and leg length discrepancy (LLD).

Patients with pathological fractures or metabolic bone disease were excluded. Similar to Van Cruchten et al., major complications in this study were defined as post-treatment problems that created a need for unscheduled admission to hospital or unplanned operative treatment, whereas minor complications were defined as problems that were treated as an outpatient or in the community [9].

All data analyses were carried out using R statistics analysis software. Initial univariate analyses were carried out looking into patient demographics and injury details. Factors affecting time to union (full/partial weight bearing and radiologic union in three cortices) were sought using multiple regression analysis.

## Results

A total of 138 patients who underwent treatment for femoral shaft fractures were identified from the hospital database. Fourteen were excluded due to their fractures being related to a pathological cause or a background of metabolic bone disease, leaving 124 for analysis.

As can be seen in Table 1, there were 95 males (77%) and 29 females (23%), which presented a statistically significant difference, p<0.05.

**Table 1:** Summary of descriptive statistics

Stat	N (% of total) / Mean	N (% of total) / Standard Deviation (SD)	p-value
Total	124		
Gender	Male = 95 (77%)	Female = 29 (23%)	2.2 x 10^-16^
Age	<1 – 15	Median = 7 years	n/a
Side	Right = 63 (51%)	Left = 61 (49%)	0.389
Year of injury	2005 - 2021		n/a
Pattern	Spiral = 69 (56%)	Transverse = 41 (33%)	2.2 x 10^-16^
	Oblique = 11 (9%)	Butterfly = 3 (2%)	
Length stable/unstable	Unstable = 83 (67%)	Stable = 41 (33%)	8.076 x 10^-10^
Location	Proximal third = 33 (27%)		2.3 x 10^-12^
	Middle third = 80 (64%)		
	Distal third = 11 (9%)		
Pre-op Translation	Mean = 74.1%	SD = 36.2%	n/a
Pre-op AP plane angulation	Mean = 8.0° varus	SD = 12.6°	n/a
Pre-op Sagittal plane angulation	Mean = 2.9° anterior	SD = 12.4°	n/a
Pre-op Shortening	18.5 mm	SD = 16.8 mm	n/a
Comminution	Yes = 27 (22%)	No = 97 (78%)	7.8 x 10^-8^
Open	Open = 2 (2%)	Closed = 122 (98%)	2.2 x 10^-16^
Polytrauma	Polytrauma = 24 (19%)	Isolated = 100 (81%)	2.2 x 10^-16^
Operation time	Mean 82.78 minutes	SD = 64.74 minutes	n/a
Time to theatre	Mean = 5.36 days	SD = 6.09 days	n/a
Pre-op traction	Thomas = 110 (89%)	Nil = 14 (11%)	3.1 x 10^-16^
PICU stay	Yes = 9 (7%)	No = 115 (93%)	0.002
Follow-up length	Mean = 263 days	SD = 293 days	n/a
Complications	Major = 10 (8%)	Minor = 5 (4%)	n/a

**Figure 1:**
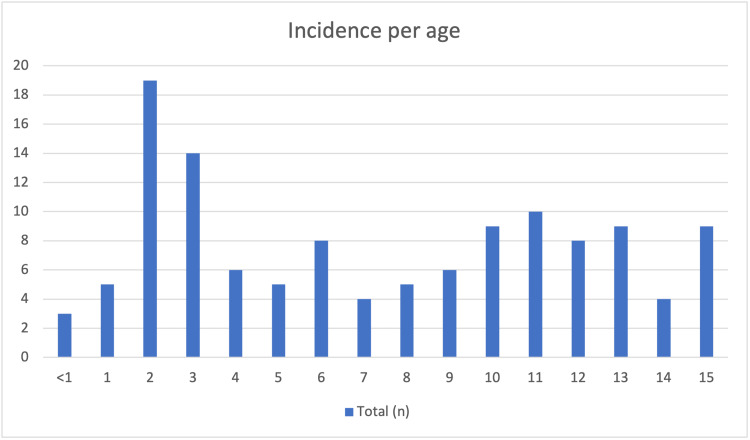
Barchart of incidence per patient age

There was a wide distribution of patients in terms of age, with the highest incidence in patients of two and three years of age, consisting 19 and 14 fractures respectively. Below (Figure 1) is a barchart detailing the incidences per patient age.

There were also a statistically significant number of spiral (69, 56%) and transverse (41, 33%) fracture patterns compared to oblique (11, 9%) and butterfly (3, 2%) fracture patterns, p<0.05. Of note, there were no segmental fractures. Length stable fracture patterns, that is fractures with a low risk of shortening, include transverse fractures, whereas length unstable fracture patterns have a high risk of shortening and include spiral, oblique, and butterfly patterns. There were significantly more length unstable than length stable fracture patterns, with 83 (67%) versus 41 (33%) respectively (p<0.0001).

There were 63 (51%) right-sided fractures and 61 (49%) left-sided fractures (p=0.3). The fractures were divided into proximal, middle and distal thirds, with 33 (27%), 80 (64%), and 11 (9%) respectively. Mean translation was 74.1% with a standard deviation (SD) of 36.2%. Mean angulation measured on AP plain film was 8.0° varus, whereas on sagittal view was 2.9° anterior, both with standard deviation of 12°. Twenty-seven (22%) of the fractures had comminution on initial plain films. Almost all of the fractures were closed, with only two (2%) open. Twenty-four (19%) of the patients were polytrauma cases.

Primary procedures included Pavlik harness, spica cast, above knee (AK) backslab, elastic stable intramedullary nails (ESIN), rigid intramedullary nails (IMN), plate fixation, and external fixation (ex-fix). The numbers of each are shown in the table below (Table 2).

**Table 2:** Primary procedures total numbers and mean age of patients AK: Above knee; ESIN: Elastic stable intramedullary nails; IMN: Intramedullary nails; Ex-fix: External fixation.

Primary Procedure	Numbers	Mean Age (SD)
Harness	1	<1
Spica	44	2.4
AK cast	1	5
ESIN	21	8.6
Plating	16	8.2
Ex-fix	14	10.9
IMN	27	12.4

**Figure 2:**
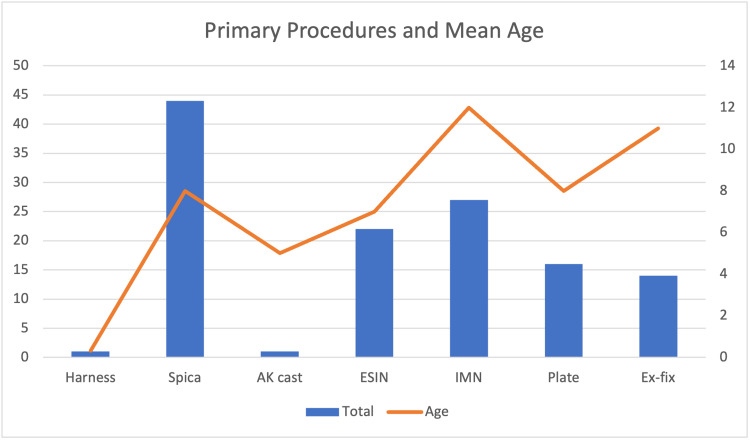
Graph showing total numbers and mean age for each primary procedure AK: Above knee; ESIN: Elastic stable intramedullary nail; IMN: Intramedullary nail; Ex-fix: External fixation,

The graph below (Figure 2) shows the total number of primary procedures carried out and the mean age at the time of presentation for each procedure.

In this cohort, external fixation was used as a definitive fixation for all cases except one, a 15-year-old polytrauma patient who underwent external fixation followed by a definitive IMN seven days later. Unfortunately due to the mechanism of a crush injury, the threatened limb became non-viable, and the patient underwent an amputation at the level of hip joint two weeks later.

**Figure 3:**
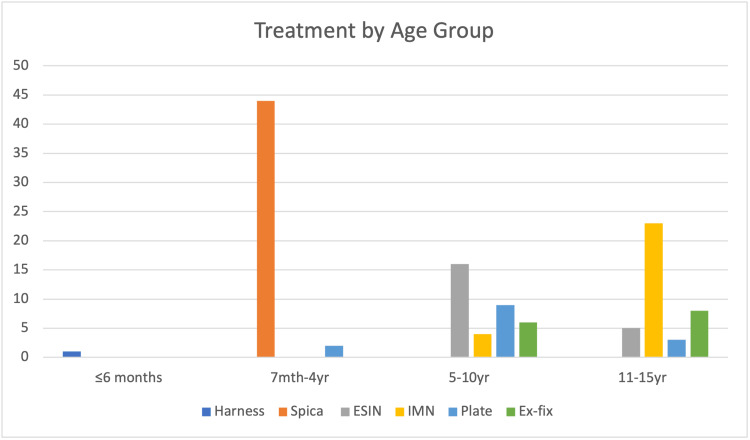
Barchart showing the primary procedures carried out by age group ESIN: Elastic stable intramedullary nail; IMN: Intramedullary nail; Ex-fix: External fixation.

The figure below (Figure 3) depicts the numbers of primary procedures carried out by age groups of ≤6 months, 7 months-4 years, 5 years-10 years, and 11 years to 15 years of age.

Pre-operative (pre-op) traction using Thomas splint was used on the majority of patients (89%). Nine (7%) patients were admitted into PICU in the perioperative period.

The mean time to theatre for all cases was 5.36 days with SD of 6.1 days. The mean time to theatre for polytrauma versus isolated cases was 1.33 days versus 6.33 days respectively, representing a statistically significant difference (p<0.0001).

The mean operation time for all procedures was 82.78 minutes, with SD of 64.74 minutes. The mean operation time for polytrauma cases was 154.25 minutes compared to 65.63 minutes for isolated cases, again showing a significant result (p=0.0001).

For patients who were discharged from care at the time of writing, the mean follow-up length was 263 days, with SD of 293 days.

As referenced in the methodology, the time to union was defined as radiological union in three cortices and full/partial weight bearing as able. For this study, the overall mean time to union was 90 days. The average time to union based on fracture location and primary procedure is shown in the table below (Table 3).

**Table 3:** Mean time to union based on primary procedure and fracture location AK: Above knee; IMN: Intramedullary nail; Ex-fix: External fixation; ESIN: Elastic stable intramedullary nail.

Primary Procedure	Time to union (days)
Harness	39
Spica	54.38
AK cast	117
ESIN	96.71
Plating	115.27
Ex-fix	96.17
IMN	128.84
Fracture location	Time to union (days)
Proximal third	123.29
Middle third	77.79
Distal third	84.1

After carrying out multiple linear regression of time to union based upon 16 different factors, the factors that affected time to union that was of statistical significance were open versus closed fracture (p<0.0001), operation time (p=0.01) and translation (p=0.04). The model accounted for approximately 42% of variance in time to union, which again was statistically significant (p<0.01).

From Table 3, we can see that mean time to union was highest for intramedullary nailing at 128 days, followed by above knee cast and plating at 117 and 115 days each. Mean time to union was lowest for Pavlik Harness at 39 days, followed closely by spica at 54 days. Elastic nailing and plating techniques both had a mean time to union of 96 days respectively. However, following multiple regression accounting for other factors, the difference in the mean time to union between primary procedures carried out was not statistically significant (p>0.05).

The mean time to union for patients with open versus closed fractures was 465 days and 83.87 days respectively (p<0.0001). The two patients who suffered open fractures were aged 14 and 15 years respectively. Both cases were managed with operative debridement and intramedullary nailing as definitive fixation.

One of the patients with open fracture went on to non-union after nine months, and was treated with exchange nailing, which was then followed by reamed exchange nailing 18 months after. The fracture then went on to successfully unite.

The mean time to union for patients with ≤20% translation versus ≥100% translation was 56.75 days versus 104.5 days.

**Figure 4:**
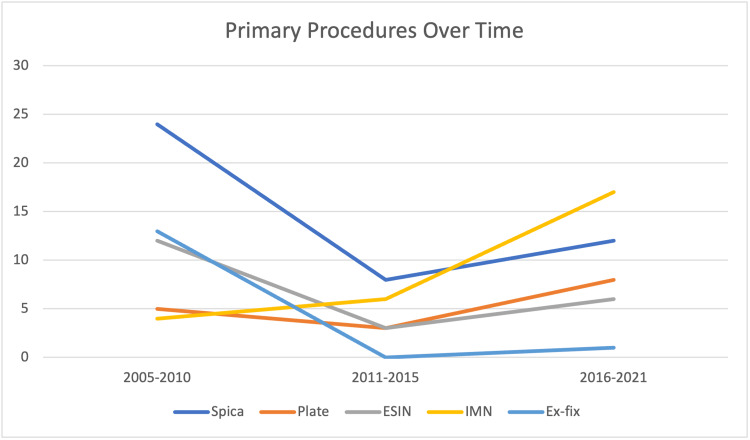
Line graph showing numbers of primary procedures carried out over time ESIN: Elastic stable intramedullary nail; IMN: Intramedullary nail; Ex-fix: External fixation.

The line graph below (Figure 4) depicts the number of primary procedures carried out in five/six-year intervals for this cohort of patients. Of particular interest within this graph is the dramatic increase in the use of intramedullary nails over the 16-year period, especially after 2011.

Complications

In this cohort of patients, there was a 4% minor complication rate, i.e., five patients who suffered complications that did not require further unplanned admission or operation. These complications included a patient with superficial wound infection following plating which was treated with oral antibiotics, and four patients who struggled with quadriceps wasting, osteopenia, knee stiffness, and symptoms of knee instability respectively. No neurovascular deficit was noted. These were all found in clinic follow-up and were managed successfully with outpatient physiotherapy. They are detailed below (Table 4).

**Table 4:** Minor complications, number of patients suffered each, and treatment ESIN: Elastic stable intramedullary nail; ROM: Range of motion.

Complications not needing repeat procedures	Numbers	Primary procedure	Treatment
Superficial infection	1	Plate	Oral antibiotics
Osteopenia	1	Plate	Physiotherapy
Symptoms of knee instability	1	Plate	Physiotherapy
Quadriceps wasting	1	ESIN	Physiotherapy
Reduced hip ROM	1	ESIN	Physiotherapy

On the other hand, there were 10 patients with complications that required further unplanned operative treatment and admission, a rate of 8%, as detailed in the table below (Table 5). Removal of metalwork was not included as this is a standard follow-up procedure for children with femoral shaft fractures in this centre.

**Table 5:** Primary procedures with complications requiring repeat procedures ESIN: Elastic stable intramedullary nail; Ex-fix: External fixation; IMN: Intramedullary nail; AP: Anteroposterior.

Primary Procedure	Repeat Procedures	Reason	Age
Spica	Spica	Unacceptable position 23 varus, 13 ant	2
Spica	Spica	21 varus, 28 ant	3
Spica	ESIN	Unacceptable position shortening 2.6 cm, AP 21 varus, Sag 0	4
Ex-fix	Ex-fix	Pin site infection	2
Ex-fix	Epiphysiodesis	2 cm femur overgrowth	7
Ex-fix	Ex-fix	Fracture at ex-fix pin	13
Ex-fix	IMN followed by amputation	Non-viable leg due to crush injury	15
ESIN	Plate	Unacceptable position, 17 varus, 12 ant	6
Plate	Bone stimulator followed by IMN	Delayed union	9
IMN	Exchange nail	Nonunion after 9 months	15

Mean leg length discrepancy (LLD) for the entire cohort was a lengthening of 0.076 cm. At the time of last follow-up, seven patients experienced shortening whereas 14 patients experienced overgrowth of the femur. Shortening ranged from 0.5 cm to 1.5 cm, whereas overgrowth ranged from 0.4 cm to 2 cm. Two patients had 2 cm LLD post-treatment, one underwent plating for fracture treatment, and the other external fixation. The patient with external fixation went on to have epiphysiodesis for correction of leg length. This was the only patient who underwent intervention for LLD.

Multiple regression was carried out on leg length discrepancy (LLD) based on 16 factors. The only factor found to be significant was the side of limb in which the fracture occurred, wherein right-sided fractures were found to be associated with shortening post-fracture treatment (p=0.024).

In terms of primary procedures, the leg length discrepancy rate was highest for diaphyseal plating with 31.3% and lowest for external fixation with 7.1%, detailed in Table 6. Mean pre-operative shortening for patients treated with plates versus external fixation was 18.88 mm versus 27.08 mm.

**Table 6:** Leg length discrepancy rate, number of cases and range of lengthening or shortening based on primary procedures and age group AK: Above knee; ESIN: Elastic stable intramedullary nail; IMN: Intramedullary nail; LLD: Leg length discrepancy.

Treatment / Age group (Total, n)	Shortening (cm)	Lengthening (cm)	LLD rate (%)	Median (Range), cm
Harness (1)	Nil	Nil	0	0 (0)
Spica (44)	3 cases: 0.5 – 1.5	3 cases: 1 – 1.5	13.6	0 (-1.5 – 1.5)
AK cast (1)	1 case: 0.5	Nil	100	-0.5 (-0.5)
ESIN (21)	Nil	2 cases: 1.3	9.5	0 (0 – 1.3)
IMN (27)	Nil	6 cases: 0.4 - 1.5	22.2	0 (0 – 1.5)
Plate (16)	3 cases: 1 – 1.5	2 cases: 0.5 – 2	31.3	0 (-1.5 – 2)
External fixation (14)	Nil	1 case: 2 cm	7.1	0 (0 – 2)
≤6 months (1)	Nil	Nil	0	0 (0)
7 months – 4 years (46)	3 cases: 0.5 – 1.5	3 cases: 1 – 1.5	13	0 (-1.5 – 1.5)
5 – 10 years (37)	4 cases: 0.5 – 1.5	3 cases: 1.3 – 2	18.9	0 (-1.5 – 2)
11-15 years (40)	Nil	8 cases: 0.4 – 2	20	0 (0 – 2)

The rate of leg length discrepancy increased with an increase in age group, however, this was not statistically significant based on multiple regression. The 11-15 years group had an incidence of eight cases of lengthening and LLD rate of 20% whereas the 7 months-4 years group had an incidence of six cases of shortening or lengthening and LLD rate of 13%.

## Discussion

In this study, the vast majority of cases were closed fractures (98%). This is in keeping with previous studies that report closed fractures of the femur are more frequently observed due to its soft tissue cover, as compared to fractures of the tibia [9]. Both open femoral fracture cases underwent initial external fixation to stabilise the bone and to allow the soft tissue to rest and recover.

Paediatric femoral fractures are one of the most common orthopaedic injuries in children that require hospitalisation [10,11], which creates a significant socioeconomic cost for the patients, their family, and for the health care service as a whole. Heyworth et al. noted that despite the cost of treatment increasing throughout their study period, it was still grossly underestimating the wider cost of managing the injury, for example, due to potential requirements for physiotherapy, missed school days for patients, and missed days of work for parents or guardians [10].

Within this cohort of 124 patients, multiple linear regression looking at factors affecting time to union found that open fractures (p<0.0001) significantly affected union time. The entire model represented approximately 42% of variance in time to union, a statistically significant amount. The mean time to union for open versus closed fractures was 465 vs 83.87 days. There were only two open fractures within this cohort, however, this demonstrates the significant morbidity that open fractures create despite adhering to current guidelines of treating with antibiotic and tetanus cover, adequate debridement and tissue coverage in an appropriate time.

Tsang et al. had looked into patients with traumatic femoral diaphyseal fracture non-union who underwent exchange nailing, and found that 30% had evidence of infection as a risk factor for non-union, and infection was associated with an increased median time to union of seven months [12]. In the two patients with open fractures there was no evidence of infection and thus reflects the significant energy of the injury.

Since the 1990s, there has been a paradigm shift in treating femoral shaft fractures in children. It had become clear that early spica casting moderately improved outcomes in children aged two to four years of age. However, for school-aged children in particular, operative fixation has increasingly become the treatment option of choice amongst surgeons as this had been shown to improve the time to return to school or activity, reduce the length of inpatient stay, and help alleviate some burden of parents or guardians with taking time away from work [13,14]. This is certainly represented in this cohort of patients, whereby operative fixation is by far the most common method of treatment in the school-attending age group of six years old and above.

This study has found that all patients aged five years old and above were treated with operative internal fixation rather than spica casting, and there were also two cases in children aged two and four years old respectively who also underwent plating fixation. In 2019 Alluri et al. found an increase in femoral shaft fracture operative fixation of 35% and 58% in children aged four and five respectively during the period of 1997 and 2012 [15].

Furthermore, Talbot et al. found that children aged four to six years old treated in major trauma centres were significantly more likely to be treated with open reduction and internal fixation compared to children treated in trauma units [5]. As this study was looking at patients treated in the Children’s Hospital for Wales, a tertiary centre in South Wales, surgeons' experience and confidence in the operative management of children’s fractures may have played a significant part.

In their 2020 guidelines, the American Association of Orthopaedic Surgeons (AAOS) found strong quality evidence (level 1 or 2) of a need to carefully review children with a femoral shaft fracture younger than 36 months for evidence of non-accidental injury [11]. This study found one case of suspected non-accidental injury in a child of four months old, which was referred to Social Services. The child suffered a midshaft spiral fracture which was treated in a Pavlik Harness without any post-operative complications or leg length discrepancy.

In the seven months to four years age group, 44 patients were treated with hip spica cast, whereas two patients were treated with plate fixation. The mean pre-operative fracture shortening for spica versus plate fixation in this group was 13.80 mm and 25 mm, respectively. The mean time to theatre for spica casting was 9.70 days, versus three days for all other treatment methods, a statistically significant difference (p=2.32 x 10^-10^). This is in keeping with current evidence whereby in-line traction with delayed spica casting may reduce the incidence and severity of malunion [16].

Between five to 10 years old, we can see that there was an increase in the variety of fixation methods used. Elastic stable intramedullary nails were the most common method utilised, followed by plate, external fixation, and rigid intramedullary nails respectively. In terms of fracture patterns in this age group, there were 15 transverse or length stable fractures, and 22 length unstable fractures. This fits with the frequency of elastic nails preferentially used in transverse or length stable fractures carried out compared to other fixation techniques, 16 ESIN and 21 other fixation methods respectively.

This age group had more elastic or rigid intramedullary nailing carried out than plating or external fixation with a relatively low complication rate, which is in keeping with the systematic review by Van Cruchten et al. [4]. Children aged two to 10 years old treated with either rigid or flexible intramedullary nails had significantly lower rates of malunion and LLD, lower means of angulation and shortening, and earlier achievement of rehabilitation milestones [4].

Femoral malrotation has not been a reported complication in this cohort of elastic intramedullary nails, however, this was not analysed beyond post-operative clinical examination and plain films in the outpatient department. Abnormal rotation was measured as more or less than two standard deviations away from the known mean femoral rotation for a particular age. Femoral malrotation has been reported to have an incidence of up to 41.6% on post-operative computed tomography (CT) scanning [17].

The overall complication rate of elastic intramedullary nails (ESIN) in this cohort of patients was 14.3%, with one major complication and two minor complications. The major complication was due to unacceptable angulation during follow-up that required a repeat procedure to plate fixation. ESIN had a low leg length discrepancy rate of 9.5%, the lowest second to external fixation. The AAOS recommend that there is strong evidence for the use of elastic nails in the five to 11-year age group [11], as it has been shown to be an effective method of fixation.

Finally, in the oldest age group of 11-15 years old, it was found that rigid intramedullary nails were by far the most common treatment method used, followed by external fixation, elastic nails and plate.

Importantly in this cohort of patients, there were two piriformis entry nails inserted in the earlier years of the study, and the rest of the rigid nails were lateral entry nails. This is an important finding as piriformis entry nails are associated with avascular necrosis and there is a reported incidence of 1-5% [18-20]. The advent of lateral entry nails was an important advance in using nails in children, as they are designed to be inserted via the lateral trochanter, avoiding injury to the blood supply. Two case series and one systematic review have reported no incidence of AVN with lateral entry nails [18, 21, 22]. In this cohort of 27 rigid intramedullary nails, there was no incidence of AVN found with either piriformis entry or lateral entry nails.

The overall complication rate of rigid intramedullary nails in this cohort was low at 3.7%, with just one major. This is an important finding as with the emergence of lateral entry nails, we are able to utilise rigid nailing techniques to manage femoral shaft fractures, with a reduction in incision length, soft tissue dissection and operative time, as we often use in adults. The youngest age that rigid nail was used was nine years old.

The change in the usage of implants over time is in keeping with the evolution of implants and surgical techniques. This can be seen with the increased usage of lateral entry or greater trochanteric entry nails with increasing evidence of good outcomes. Similarly, there had been an increase in the usage of plates in the final six-year period compared to the previous 10 years, coinciding with an obvious reduction in external fixation numbers to almost zero. This reflects the emergence of minimally-invasive plate osteosynthesis (MIPO) techniques and evidence of submuscular plating in the fixation of high-energy or comminuted fractures with positive outcomes.

Currently, there is limited evidence in the literature to support submuscular plating versus rigid nailing for femoral diaphyseal fractures, however, in this cohort of patients rigid nailing had a lower overall complication rate compared to plating (complication rate of 25% for plating).

The current recommended use of external fixation is for damage control orthopaedics, whereby a quick operation to stabilise the bony injury and soft tissue damage is needed to allow the patient time to improve physiologically. External fixation is also sometimes found in cases where patients are to be transferred elsewhere. The relatively high overall complication rate of 28.6% in this cohort of patients supports this limited use of external fixation, and this is demonstrated by the significant drop in its usage over the study time.

Weaknesses of this study are due to it being retrospective in nature, the data collected are reliant on clinical letters or notes leaving it susceptible to human error. Although an effort was made to control for confounders using multiple linear regression, a potential limitation in control of unknown exposure factors still exists.

## Conclusions

This was a pragmatic study that explored paediatric diaphyseal femoral fractures in a single tertiary centre, the change in management over time, overall outcomes and complications, variables affecting time to union and leg length discrepancy, and management per age group. The findings in this study are in keeping with the latest trends for the treatment of femoral shaft fractures in children. Following multiple linear regression with 16 possible explanatory factors, open fractures, pre-operative translation and operation time were found to affect time to union to a statistically significant degree, whereas the side of fracture was found to significantly affect leg length discrepancy. Time to union was highest for IMN and lowest for Pavlik Harness, however, after accounting for multiple covariates this was not a statistically significant finding.

Operative fixation was carried out in patients as young as two years of age, and was the management of choice in children five years old and above. Complication rates were found to be very low for rigid intramedullary nailing, with no cases of avascular necrosis, whereas complication rates for plate fixation were found to be much higher. Finally, there was a significant increase in the utilisation of intramedullary nailing and submuscular plating over the 16 years coinciding with a noticeable decrease in the usage of external fixation for definitive treatment of femoral diaphyseal fractures in children.
